# Posterior reversible encephalopathy syndrome as a rare presentation of systemic lupus erythematosus: a case report

**DOI:** 10.1097/MS9.0000000000005055

**Published:** 2026-05-07

**Authors:** Surya Prakash Joshi, Sanjeev Kharel, Ramesh Balayar, Sachet Subedi, Prasanna Karki, Kamal Prakash Saud, Saket Jha

**Affiliations:** a Maharajgunj Medical Campus, Institute of Medicine, Maharajgunj, Kathmandu, Nepal; b Department of Internal Medicine, Tribhuvan University Teaching Hospital, Maharajgunj, Kathmandu, Nepal

**Keywords:** posterior reversible encephalopathy syndrome, seizures, systemic lupus erythematosus

## Abstract

**Introduction and importance:**

Posterior reversible encephalopathy syndrome (PRES) is a rare clinical-radiological entity characterized by the rapid onset of neurological symptoms accompanied by typical radiological signs of vasogenic edema predominantly in the posterior brain region. Numerous causes can contribute to PRES, including hypertension, renal failure, pre-eclampsia, and immunosuppression. Systemic lupus erythematosus (SLE) is a multisystem autoimmune disorder involving several immune dysfunctions and can affect the central nervous system. Although rare, cases of PRES have been documented in individuals diagnosed with SLE.

**Case presentation:**

We here describe a case of an 18-year-old female who was newly diagnosed with SLE and experienced generalized tonic and clonic seizures during treatment as an inpatient in the hospital. On further evaluation for the cause of the seizure, the presence of PRES was found in a brain MRI. The patient condition was managed with antiepileptic medication and kept on regular BP monitoring. For the management of SLE, due to infertility risk, the patient was discharged on mycophenolate mofetil.

**Clinical discussion:**

PRES is an uncommon but recognized complication in SLE, often triggered by hypertension, immunosuppressive therapy, or disease activity. MRI remains the diagnostic gold standard, revealing characteristic posterior white matter changes. Early recognition and supportive management, including seizure control, can lead to complete recovery and prevent long-term deficits.

**Conclusion:**

PRES, though rare in SLE, should be considered in patients with seizures, altered mental status, and visual disturbances with supportive brain imaging. Further research is needed to clarify its mechanisms and improve management.

## Introduction and importance

Posterior reversible encephalopathy syndrome (PRES) is an uncommon neurological disorder characterized by its clinical symptoms and distinctive appearance on magnetic resonance imaging (MRI)^[^1^]^. In 1996, Hinchey *et al* initially reported PRES as a clinical illness with a variety of manifestations but with a shared radiological feature of posterior cerebral cortex vasogenic edema^[^2^]^. Systemic lupus erythematosus (SLE) is a multisystemic autoimmune disorder marked by several immunological dysfunctions and is capable of affecting the central nervous system (CNS)^[^3^]^. Patients with SLE have a prevalence of PRES ranging from 0.7 to 1.4%^[^4^]^. Headache, vomiting, changed mental status, abnormalities in vision, seizures, unconsciousness, and focal neurological impairments are the typical symptoms of PRES^[^5^]^. This manuscript complies with the TITAN Guidelines 2025 for the responsible and transparent use of Artificial Intelligence in academic publishing^[^6^]^.

HIGHLIGHTS

- Though rare, posterior reversible encephalopathy syndrome (PRES) should be considered in systemic lupus erythematosus (SLE) patients presenting with sudden neurological symptoms.
- MRI is used to confirm the diagnosis of PRES, showing typical parieto-occipital vasogenic edema.
- Treatment of PRES is supportive, along with regular monitoring of blood pressure.

Following Case Report (CARE) guidelines, in this report, we describe a case of PRES in an 18-year-old female with SLE^[^7^]^.

## Case presentation

An 18-year-old female presented in the emergency department with chief complaints about fever for 10 days and yellowish discoloration of the body for 7 days. The fever was an insidious onset, continuous, with a maximum recorded temperature of 102 °F, associated with chills and rigors, and relieved after taking antipyretics. The yellowish discoloration was initially on the sclera and later progressed to the whole body. It was associated with high-colored urine but not with itching and clay-colored stool. She also complained of multiple painful oral ulcers for the same duration. She gives a history of symmetrical inflammatory polyarthritis involving the small joints of the hands and the knee joints for 6 months. She also reports a history of excessive fatigue for the past 3 months. Her bowel and bladder habits were normal. She did not have any prior medical or surgical history. She did not complain about frothy urine, red urine, shortness of breath, palpitations, cough, or chest pain.

On examination, she was febrile with a temperature of 101 °F, blood pressure (BP) was 145/70 mm Hg. Icterus was present. The erythematous rash was present over the malar region of the face and body, along with multiple oral ulcers over the lips and buccal cavity. On per-abdomen examination, there was tenderness over the epigastric region, but there was no palpable mass. On chest auscultation, crepitations were present over the bilateral infrascapular regions. Examination of all other systems was grossly intact.

She was evaluated and managed in a line of fever and jaundice. On blood investigation, she had bi-cytopenia with hemoglobin of 8.6 gm% and platelet count of 130 000 /µl. She had a direct coombs test positive (3+) and low MCV with peripheral blood smear showing normocytic normochromic RBCs along with microcytic hypochromic RBCs and occasional pencil cells. On urine analysis, she had plenty of RBCs with albumin 1+, which on quantification, amounts to 1.287 gm in 24 hours. She had conjugated hyperbilirubinemia with a total bilirubin of 124 µmol/l and direct bilirubin of 58 µmol/l. She had transaminitis with SGOT raised to 177 U/l and SGPT raised to 91 U/l. On ultrasonography, mild fluid collection was seen in the abdomen, pelvis, and pleural cavity. The gall bladder was collapsed with diffuse edematous wall thickening measuring up to 12 mm. Echocardiography was also done, which shows mild pericardial effusion with normal left ventricle and diastolic function.

On the background of relevant clinical details, bi-cytopenia, serositis, and proteinuria, a workup was done for connective tissue disease, and she was found to have SLE as per laboratory investigations given in Table 1.

**Table 1:** Laboratory blood investigations.

S.N.	Blood parameters	Obtained value	Reference range
1.	C-reactive protein (CRP)	96 mg/l	< 10 mg/l
2.	ANA	Positive	
3.	Anti dsDNA	524.73 IU/ml	<100 IU/ml
4.	C3	26.5 mg/dl	90–180 mg/dl
5.	C4	1.5 mg/dl	10–40 mg/dl
6.	Nucleosomes	30	26–50 (Moderately positive)
7.	Histone	14	11–25 (Positive)
3	Lupus anticoagulant Ratio	1	<1.3
4.	Anti-cardiolipin antibody IgG	5.88 GPL	<15.00 GPL
5.	Anti-cardiolipin antibody IgM	4.07 GPL	<12.50 GPL
6.	Anti-SSA, anti-SSB, & anti-Jo	Negative	

**Figure 1.**
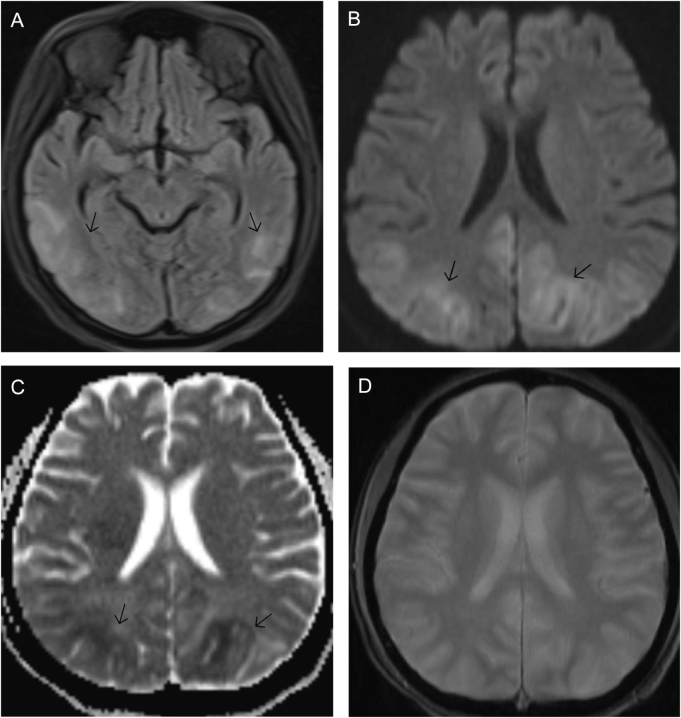
(A) Axial T2-FLAIR MRI of the brain at the level of the basal ganglia and temporal lobes demonstrates bilateral cortical and subcortical hyperintensities involving the posterior temporal and occipital regions (arrows), consistent with edematous changes. (B) Axial diffusion-weighted imaging (DWI) at the level of the posterior horns of the lateral ventricles shows bilateral hyperintense signal in the occipital lobes (arrows), indicating restricted diffusion. (C) Corresponding axial apparent diffusion coefficient (ADC) map at the same level demonstrates low ADC signal in the bilateral occipital regions (arrows), confirming true diffusion restriction. (D) Axial gradient echo (GRE) image at the corresponding ventricular level shows no blooming artefact.

Once the diagnosis of SLE was made, she was planned to manage with intravenous methylprednisolone. However, during the second day of treatment, the patient developed a sudden onset of headache, blurred vision, and generalized tonic-clonic seizures. Her condition was managed with antiepileptic and analgesic medications. Her BP was 130/86 mm Hg. The patient was further evaluated for the cause of the seizure. A lumbar puncture was performed for cerebrospinal fluid (CSF) examination. Cerebrospinal fluid analysis showed an opening pressure of 9 cm H₂O, WBC count of 2 × 10^6^ cells/l, protein concentration of 0.23 g/l, glucose level of 2.9 mmol/l (corresponding serum glucose level at 5.2 mmol/l), and negative Gram stain and culture. Additionally, a contrast-enhanced computed tomography of the brain was conducted and found to be normal as well. On MRI brain, cortical and subcortical T2/FLAIR high signal intensity was observed in the bilateral cerebral hemispheres, predominantly in the occipital lobe (Fig. 1A). Diffusion-weighted imaging revealed corresponding hyperintense signals in the bilateral occipital lobes (Fig. 1B). The associated low signal on apparent diffusion coefficient (ADC) mapping confirmed true diffusion restriction in these areas (Fig. 1C). No evidence of hemorrhage was observed on gradient echo imaging (Fig. 1D). The overall radiological pattern was consistent with PRES with focal diffusion restriction. The patient was kept on regular BP monitoring and under antiepileptic medication. Serial BP recordings and renal parameters around the time of neurological deterioration are summarized in Table 2.

**Table 2:** Clinical and laboratory parameters around onset of PRES.

Hospital day	BP (mm Hg)	Serum creatinine (µmol/l)	Urine protein	Neurological status
Day 1	145/70	74	1.287 g/24 h	No neurological symptoms
Day 2	134/84	-	-	No neurological symptoms
Day 3 (Pre-seizure)	126/82	-	-	Headache
Day 3 (Post-seizure)	130/86	72	-	Generalized tonic–clonic seizure

**Figure 2.**
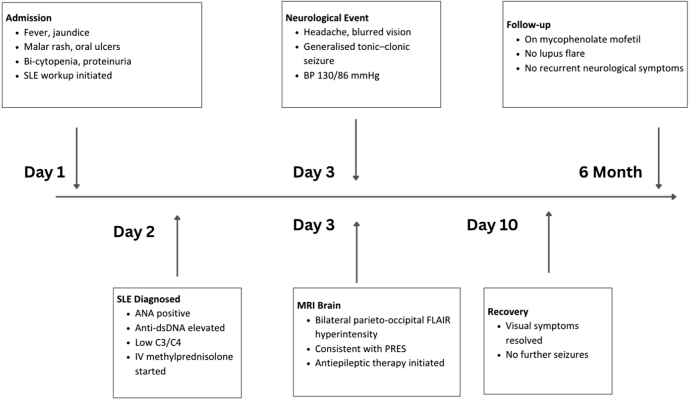
Timeline of clinical course.

After a week, her vision recovered. For the management of SLE, the patient was counseled for cyclophosphamide initially, but due to the risk of infertility, the patient deferred it. Then she was kept on mycophenolate mofetil and discharged. The patient has been under follow-up for 6 months without experiencing any significant lupus exacerbations (Fig. 2).

## Clinical discussion

PRES is a syndrome marked by temporary neurological symptoms like headache, seizures, altered mental state, and vision issues, accompanied by typical radiological signs of vasogenic edema in the posterior brain region^[^8^]^. The most common causes of PRES are autoimmune diseases, eclampsia, hypertension, drug abuse, and kidney illness^[^9^]^. The exact cause of PRES in SLE is not fully understood. Traditionally, it is thought to occur when a sudden increase in BP surpasses the brain’s autoregulation, causing brain hyperperfusion injury. Conversely, an exaggerated autoregulatory response might lead to cerebral vasoconstriction and ischemia, however hypertension alone does not fully explain PRES^[^10^]^. Immunosuppressive drugs like high-dose corticosteroids and cyclosporine have been associated with the development of PRES^[^11^]^. PRES has been reported during high-dose steroid therapy, even in the absence of arterial hypertension, which is a well-recognized side effect of corticosteroid use^[^12^]^. Our patient was also undergoing treatment with methylprednisolone for SLE.

A review done by Liu *et al* showed SLE-PRES in 1.4% of all SLE consultations, with all patients being female and having a mean onset age of 22.93 ± 2.48 years^[^13^]^. In a study conducted by Fugate *et al*, it was noted that among 113 patients with PRES, seizures were the most frequently observed symptom, followed by encephalopathy, headaches, and visual disturbances^[^14^]^. Over 70% of PRES patients have hypertension, whereas a considerable number have BP levels within normal ranges or only slightly elevated^[^15^]^. Our patient’s BP was regularly monitored throughout the entire hospital admission, and no episodes of elevated BP were noted. Renal function was stable, and there was no evidence of acute kidney injury at the time of neurological deterioration. While baseline proteinuria was present in keeping with active lupus, there was no documented acute worsening. In this clinical context, active SLE is the most plausible contributor to the development of PRES in our patient.

Differential diagnosis for PRES in a patient with a pre-established diagnosis of SLE includes neuropsychiatric-lupus, lupus cerebritis, meningitis, ischemia, a primary seizure disorder, hemorrhage, and many others, and PRES should be considered an elimination paraphrase with supportive findings^[^16^]^. CSF analysis showed no pleocytosis or biochemical abnormality, making infectious etiologies unlikely. Neuropsychiatric-lupus was ruled out due to the absence of psychiatric symptoms and normal CSF findings. Metabolic causes were excluded based on normal electrolyte and glucose levels. Brain imaging plays a central role in diagnosing PRES and excluding other potential diagnoses out of which MRI of the head is typically the preferred imaging modality. The traditional MRI findings initially depicted bilateral subcortical hyperintense regions affecting the occipital and parietal lobes. However, subsequent reports have indicated the involvement of additional lobes, including the cerebellum^[^17^]^. In our patient, MRI revealed bilateral cortical and subcortical hyperintensities on T2-FLAIR sequences, predominantly in the occipital lobes. Diffusion-weighted imaging demonstrated corresponding hyperintense signals in these regions, with associated low signal on ADC mapping, confirming true diffusion restriction, suggesting PRES. Vascular imaging (MRA/MRV) was not performed, as there were no clinical or radiological features suggestive of venous sinus thrombosis.

As our patient was of young age with concerns regarding infertility, cyclophosphamide was deferred due to its known gonadotoxic side effects^[^18^]^. Mycophenolate is an alternative immunosuppressant for lupus nephritis and systemic disease activity while carrying low risk of infertility compared to alkylating agents^[^19^]^. In our case, this approach allowed for the control of systemic SLE without recurrence of neurological symptoms during follow-up.

Treatment of PRES is supportive, addressing the underlying causes and effectively managing related complications like seizures^[^20^]^. Similarly, our patient was managed with antiepileptics and regular BP monitoring. PRES is regarded as a benign condition, and full recovery is anticipated. However, residual neurological deficits have been documented in 10–20% of cases^[^21,22^]^.

## Conclusion

For individuals with SLE, early detection and treatment of PRES is crucial. PRES should be kept in consideration while making a differential diagnosis for patients experiencing seizures, changes in mental state, and vision disturbances consistent with brain imaging, even though it is uncommon in SLE, especially when undergoing immunosuppressive treatment like methylprednisolone. Further studies are needed to elucidate the underlying mechanisms and optimize treatment strategies for PRES in the context of SLE.

## Data Availability

All data and materials used in this editorial are derived from publicly accessible sources, including peer-reviewed articles, and referenced scientific literature. Full citations are provided within the text to ensure transparency and facilitate further exploration.

## References

[1] García-Grimshaw M Domínguez-Moreno R Valdés-Ferrer SI. Posterior reversible encephalopathy syndrome: an underrecognized manifestation of systemic lupus erythematosus. Neurohospitalist. Published online November 25, 2019. doi:10.1177/1941874419889467. PMC7271615 32549950

[2] Hinchey J Chaves C Appignani B. A reversible posterior leukoencephalopathy syndrome. N Engl J Med 1996;334:494–500. 8559202 10.1056/NEJM199602223340803

[3] Ota Y Srinivasan A Capizzano AA. Central nervous system systemic lupus erythematosus: pathophysiologic, clinical, and imaging features. Radiographics 2022;42:212–32. 34990324 10.1148/rg.210045

[4] Liu Y Wang L Zhang X. Clinical features and outcomes of posterior reversible encephalopathy syndrome in patients with systemic lupus erythematosus. Arthritis Care Res (Hoboken). 2018;70:679–85. 23687067 10.1002/acr.22047

[5] Gatla N Annapureddy N Sequeira W. Posterior reversible encephalopathy syndrome in systemic lupus erythematosus. J Clin Rheumatol 2013;19:334–40. 23965484 10.1097/RHU.0b013e3182a21ffd

[6] Transparency In The reporting of Artificial INtelligence – the TITAN guideline. Premier Science. May 23, 2025. Accessed August 6, 2025. https://premierscience.com/pjs-25-950/

[7] Riley DS Barber MS Kienle GS. CARE guidelines for case reports: explanation and elaboration document. J Clin Epidemiol 2017;89:218–35. 28529185 10.1016/j.jclinepi.2017.04.026

[8] Khalid Rafat W Sarmast ST Shiza ST. Posterior reversible encephalopathy in undiagnosed systemic lupus erythematosus: a rare case report. Cureus 2021;13:e16945. 34513513 10.7759/cureus.16945 PMC8418817

[9] Kalaiselvan MS Renuka MK Arunkumar AS. Clinical features and outcomes of patients with posterior reversible encephalopathy syndrome. Indian J Crit Care Med 2017;21:453–56. 28808366 10.4103/ijccm.IJCCM_79_17 PMC5538094

[10] Sevilleja DA. Posterior reversible encephalopathy in systemic lupus erythematosus: a rare case report. J Neurol Neurophysiol 2021;12:1–3.

[11] Zhang L Xu J. Posterior reversible encephalopathy syndrome (PRES) attributed to mycophenolate mofetil during the management of SLE: a case report and review. Am J Clin Exp Immunol 2018;7:1–7. 29531864 PMC5840284

[12] Jabrane M Lahcen ZA Fadili W. A case of PRES in an active lupus nephritis patient after treatment of corticosteroid and cyclophosphamide. Rheumatol Int 2014;35:935–38. 25387825 10.1007/s00296-014-3173-1

[13] Liu B Zhang X Zhang FC. Posterior reversible encephalopathy syndrome could be an underestimated variant of “reversible neurological deficits” in Systemic Lupus Erythematosus. BMC Neurol 2012;12:152. 23217201 10.1186/1471-2377-12-152 PMC3545963

[14] Fugate JE Claassen DO Cloft HJ. Posterior reversible encephalopathy syndrome: associated clinical and radiologic findings. Mayo Clin Proc 2010;85:427–32. 20435835 10.4065/mcp.2009.0590 PMC2861971

[15] Hobson EV Craven I Blank SC. Posterior reversible encephalopathy syndrome: a truly treatable neurologic illness. Perit Dial Int 2012;32:590–94. 23212858 10.3747/pdi.2012.00152 PMC3524908

[16] Afilal I Nasri S Bendaoud M. Fatal outcome of posterior reversible encephalopathy syndrome (PRES) in a lupus nephropathy patient: a case report. Radiol Case Rep 2022;17:2215–19. 35496746 10.1016/j.radcr.2022.03.084 PMC9048052

[17] Lamy C Oppenheim C Méder JF. Neuroimaging in posterior reversible encephalopathy syndrome. J Neuroimaging 2004;14:89–96. 15095552

[18] Andreoli L Bertsias GK Agmon-Levin N. EULAR recommendations for women’s health and the management of family planning, assisted reproduction, pregnancy and menopause in patients with systemic lupus erythematosus and/or antiphospholipid syndrome. Ann Rheum Dis 2017;76:476–85. 27457513 10.1136/annrheumdis-2016-209770 PMC5446003

[19] Appel GB Contreras G Dooley MA. Mycophenolate mofetil versus cyclophosphamide for induction treatment of lupus nephritis. J Am Soc Nephrol 2009;20:1103–12. 19369404 10.1681/ASN.2008101028 PMC2678035

[20] Sudulagunta SR Sodalagunta MB Kumbhat M. Posterior reversible encephalopathy syndrome(PRES). Oxf Med Case Reports 2017;2017:omx011. 28473920 10.1093/omcr/omx011 PMC5410886

[21] Liman TG Bohner G Heuschmann PU. The clinical and radiological spectrum of posterior reversible encephalopathy syndrome: the retrospective Berlin PRES study. J Neurol 2012;259:155–64. 21717193 10.1007/s00415-011-6152-4

[22] Postma IR Bouma A Ankersmit IF. Neurocognitive functioning following preeclampsia and eclampsia: a long-term follow-up study. Am J Obstet Gynecol 2014;211:37.e1–e9. 10.1016/j.ajog.2014.01.042 24495666

